# Migraine and pregnancy-related headaches as a risk factor for cardiovascular and cerebrovascular events in pregnancy: a systematic review and meta-analysis of over 94 million pregnancies

**DOI:** 10.1186/s10194-025-02190-1

**Published:** 2025-11-13

**Authors:** Mohamed I. Mohamed, Rana Sameh, Michael Salib, Reem H. Ismail, Nada M. Badawy, Mona A. F. Nada

**Affiliations:** 1 Faculty of Medicine, Alexandria University, Champollion Street, Al Mesallah Sharq, Al Attarin, Alexandria Governorate, Alexandria, 5372066 Egypt; 2 Research Support Division, Alexandria Students’ Scientific Association, Faculty of Medicine, Alexandria University, Alexandria, Egypt; 3 Department of Neurology, Faculty of Medicine, Cairo University, Cairo, Egypt

**Keywords:** Migraine, Pregnancy, Stroke, Cardiovascular disease, Cerebrovascular disease, Headache, Myocardial infarction

## Abstract

**Background:**

Migraine is prevalent among women of childbearing age and is associated with increased long-term cardiovascular and cerebrovascular risk. Pregnancy, a hypercoagulable state, may potentiate these risks. This study aimed to quantify the association between migraine or pregnancy-related headaches and cerebrovascular and cardiovascular events during pregnancy and the postpartum period.

**Methods:**

We conducted a PRISMA-compliant systematic review and meta-analysis of observational studies comparing pregnant women with and without migraine or pregnancy-related headaches. PubMed, Scopus, and Web of Science were searched through May 8, 2025. Adjusted odds ratios (ORs) were pooled using random-effects models.

**Results:**

Twelve studies encompassing 94,195,776 pregnancies met the inclusion criteria. Migraine was associated with markedly increased odds of all strokes and transient ischemic attacks (OR 10.45; 95% CI 4.27–25.57) and ischemic stroke (OR 7.14; 95% CI 2.51–20.31). Hemorrhagic stroke risk was elevated but not statistically significant overall (OR 2.25; 95% CI 0.99–5.18), while subarachnoid hemorrhage showed a 69% increased odds. Regarding cardiovascular events: myocardial infarction risk increased by 96%, peripartum cardiomyopathy odds were 2.68-fold (95% CI 1.73–4.14), and spontaneous coronary artery dissection odds were 9.21-fold higher (95% CI 3.72–22.82). All included studies were rated as “good” quality by the Newcastle-Ottawa Scale.

**Conclusions:**

Migraine and pregnancy-related headaches are independent risk factors for a broad spectrum of cerebrovascular and cardiovascular events during pregnancy and the puerperium. These findings highlight the need for heightened clinical surveillance, targeted cardiovascular risk counselling, and multidisciplinary management strategies for this population.

**Supplementary Information:**

The online version contains supplementary material available at 10.1186/s10194-025-02190-1.

## Graphical Abstract

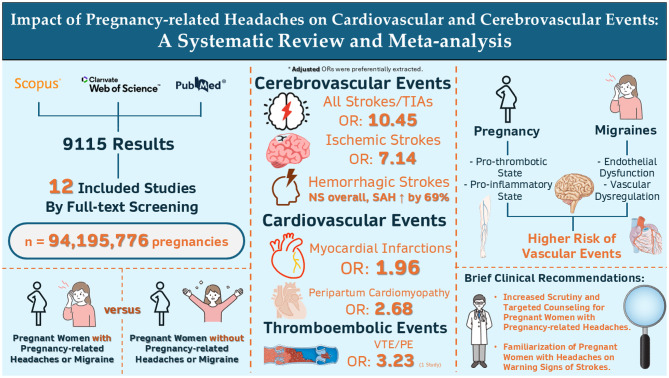

## Introduction

Migraine is the most common neurological disorder encountered in primary care settings and represents a major global health burden. According to the most recent Global Burden of Disease study, migraine ranks as the second leading cause of disability worldwide and the leading cause among young women [1, 2]. Importantly, migraine is increasingly being recognized as a “heterogeneous” condition with several different molecular and genetic bases, ultimately requiring the close integration of physicians from different specialties to offer brain health promotion and ensure a tailored approach to each patient [3].

Given its high prevalence and disabling nature, elucidating the potential systemic risks of migraine is of considerable clinical importance. Multiple case-control and cohort studies have demonstrated that individuals with migraine, or a history of migraine, have an elevated risk of stroke [4, 5].

In a large-scale systematic review of 1,152,407 participants, migraine was associated with increased long-term risks of both ischemic and hemorrhagic stroke (adjusted HR 1.41, 95% CI 1.25–1.61), myocardial infarction (adjusted HR 1.23, 95% CI 1.03–1.43), and major adverse cardiovascular and cerebrovascular events (MACCE) (adjusted HR 1.42, 95% CI 1.26–1.60). However, substantial heterogeneity was noted across outcomes, partly attributable to the presence of aura, which has been identified as an effect modifier for stroke risk [6]. Of interest, women with migraine headaches, particularly those in the reproductive period, are highly susceptible to the cardiovascular comorbidities associated with migraine. To illustrate this, a UK Biobank-based investigation of 24,038 cardiovascular events and spanning 12.9 years, compared the atherosclerotic cardiovascular disease (ASCVD) risk in women with versus without migraine. Staggeringly, an increased hazard ratio of 1.6 (95% CI: 1.16–2.2) in a multivariate adjusted model highlighted how women below 45 years of age are placed at a significantly elevated risk of ASCVD. In contrast, those above 45 years of age and with migraine demonstrated no significant association with ASCVD risk [7].

Pregnancy represents a physiologic state of heightened thrombotic risk due to marked hemostatic changes, including increased concentrations of fibrinogen, factors VII, VIII, IX, X, XII, and von Willebrand factor. This hypercoagulable state predisposes pregnant women to thrombosis [8]. The coexistence of migraine and pregnancy may therefore amplify the risk of cerebrovascular and cardiovascular complications beyond that observed in either condition alone.

A 2014 systematic review specifically examining vascular outcomes in pregnant migraineurs found strong associations between migraine and ischemic stroke in pregnancy (OR range 7.9–30.7), particularly in women with active migraine. Migraine was also linked to increased risks of acute myocardial infarction (OR 4.9, 95% CI 1.7–14.2) and thromboembolic events, including deep vein thrombosis (OR 2.4, 95% CI 1.3–4.2) and pulmonary embolism (OR 3.1, 95% CI 1.7–5.6)^9^.

Although previous systematic reviews and meta-analyses have examined migraine in relation to vascular outcomes in the general population [6, 10, 11], few have specifically addressed pregnant women. Other analyses have explored migraine in the context of adverse pregnancy outcomes [12, 13], but have not comprehensively evaluated the risk of stroke and other major cardiovascular and cerebrovascular events during pregnancy and the postpartum period.

Accordingly, the present study aimed to conduct a comprehensive meta-analysis of recent case-control and cohort studies to quantify the association between migraine (and pregnancy-related headaches) and the risk of ischemic stroke, hemorrhagic stroke, transient ischemic attack, myocardial infarction, peripartum cardiomyopathy, thromboembolic events, and arterial dissection during pregnancy and the puerperium.

## Methods

The following systematic review was conducted in accordance with the Preferred Reporting Items for Systematic Reviews and Meta-analyses (PRISMA) guidelines [14] for reproducibility and reliability. The protocol was prospectively registered with PROSPERO (ID: CRD42024607723).

### Search strategy and screening

The following databases were searched from inception until May 8, 2025, to ensure the inclusion of all relevant articles: PubMed, Scopus, and Web of Science. Search strategies included synonyms of the words: Migraine, pregnant, myocardial infarction, and stroke. No linguistic or publication period restrictions were applied. To ensure a comprehensive approach, we manually screened the references of included studies and previous systematic reviews to scope for any missing studies. For the exhaustive search strategy used, refer to the supplementary file.

For the subsequent screening process, Covidence [15], a platform designed to streamline systematic review workflows, was used. Relevant exports from the databases mentioned above were uploaded onto Covidence on May 8th, 2025. Initially, duplicates were removed by Covidence before the title and abstract screening process commenced, where two independent reviewers cast their vote on the articles according to the eligibility criteria mentioned below. Following this, potentially relevant articles were sought for retrieval for full-text screening. When conflicts arose, a third independent reviewer was responsible for resolving them as soon as they occurred. While screening, if we faced two studies published by the same authors and using data from the same source, we included only the study with the greatest sample size to prevent the duplication of data.

### Inclusion criteria

1. We considered studies eligible if they compared pregnant women with a history of either migraine (with or without aura) or pregnancy-related headaches to pregnant women without a history of migraine or pregnancy-related headaches and reported any cerebrovascular or cardiovascular outcomes including; stroke (ischemic or hemorrhagic), transient ischemic attacks (TIAs), myocardial infarction, peripartum cardiomyopathy, arterial dissection, and thromboembolic events.
2. Included study designs: Cohorts (prospective or retrospective) and case-control studies.

### Exclusion criteria

1. Studies reporting on the occurrence of adverse pregnancy outcomes, the occurrence of vascular/cardiac events without specifying the impact of migraine or pregnancy-related headaches, and the impact of post-partum headaches on vascular events were all excluded.
2. Excluded study designs: case reports, meta-analyses, reviews, animal studies, and cell-based studies.

### Data extraction

Relevant data were extracted into three independent Google Sheets tables: Baseline, Summary of Findings, and Extraction spreadsheets. Each reviewer was tasked with extracting data from a given number of articles. Subsequently, a second independent reviewer compared the extracted data to the original study to ensure accuracy. Any discrepancies were resolved by discussion.

The baseline characteristics table (seen below) included key information about the study and the population examined: Last name of the first author and year of publication; country of study; sample size; the age of the population; parity; race and ethnicity; body mass index (BMI) in kg/m^2^; additional gestational comorbidities; previous stroke status; behaviors like smoking, alcohol and drug use; and follow-up duration.

In the extraction sheet, outcomes of interest were the odds ratio (OR) and the 95% confidence intervals (CI) of the following events occurring in pregnant women with migraine/pregnancy-related headaches compared to those without migraine/pregnancy-related headaches: strokes (ischemic or hemorrhagic), hemorrhages (intracerebral - ICH and subarachnoid - SAH), myocardial infarction, peripartum cardiomyopathies, thromboembolic events, and arterial dissection. Supplementary files were also examined to look for any relevant data not reported in the main manuscript.

In the summary of findings table, the varying corresponding effect estimates along with the variables they were adjusted for were reported. This is to allow for the accuracy of interpretation of the results and to highlight the heterogeneity across the studies.

### Quality assessment

We employed the Newcastle–Ottawa Scale (NOS) [16] for Quality assessment (QA), which was adapted for cohort and case-control studies. The NOS scale evaluates each study using a point system based on the results from three domains: Selection, comparability, and outcomes. This scale assigns a total score ranging from 0 to 9 points to assess the quality of the study. An independent member conducted the QA for each study, with another independent member conducting their own assessment, and discrepancies were settled.

In the QA table, we converted NOS to AHRQ standards (good, fair, and poor quality studies) following these thresholds: Good quality: 3 or 4 stars in selection domain AND 1 or 2 stars in comparability domain AND 2 or 3 stars in outcome/exposure domain, fair quality: 2 stars in selection domain AND 1 or 2 stars in comparability domain AND 2 or 3 stars in outcome/exposure domain, and poor quality: 0 or 1 star in selection domain OR 0 stars in comparability domain OR 0 or 1 stars in outcome/exposure domain.

### Statistical analysis

The R programming software [17] was utilized to conduct the meta-analysis using the metagen function. Odds ratios (OR) were pooled alongside the 95% confidence intervals. Whenever raw data for events and total were reported in studies, we used a 2 × 2 contingency table to calculate the unadjusted OR. Whenever a study reported unadjusted and adjusted ORs, we preferentially extracted and pooled the adjusted ORs for the largest number of variables. If ORs were reported for different time periods, we extracted the OR corresponding to the latest reported time period. We conducted subgroup analyses based on the adjustment status of ORs. Whenever significant heterogeneity (p-value < 0.05 and I^2^ >50%) was encountered, we used a random-effects model to pool the ORs.

## Results

### Screening outcome

**Fig. 1:**
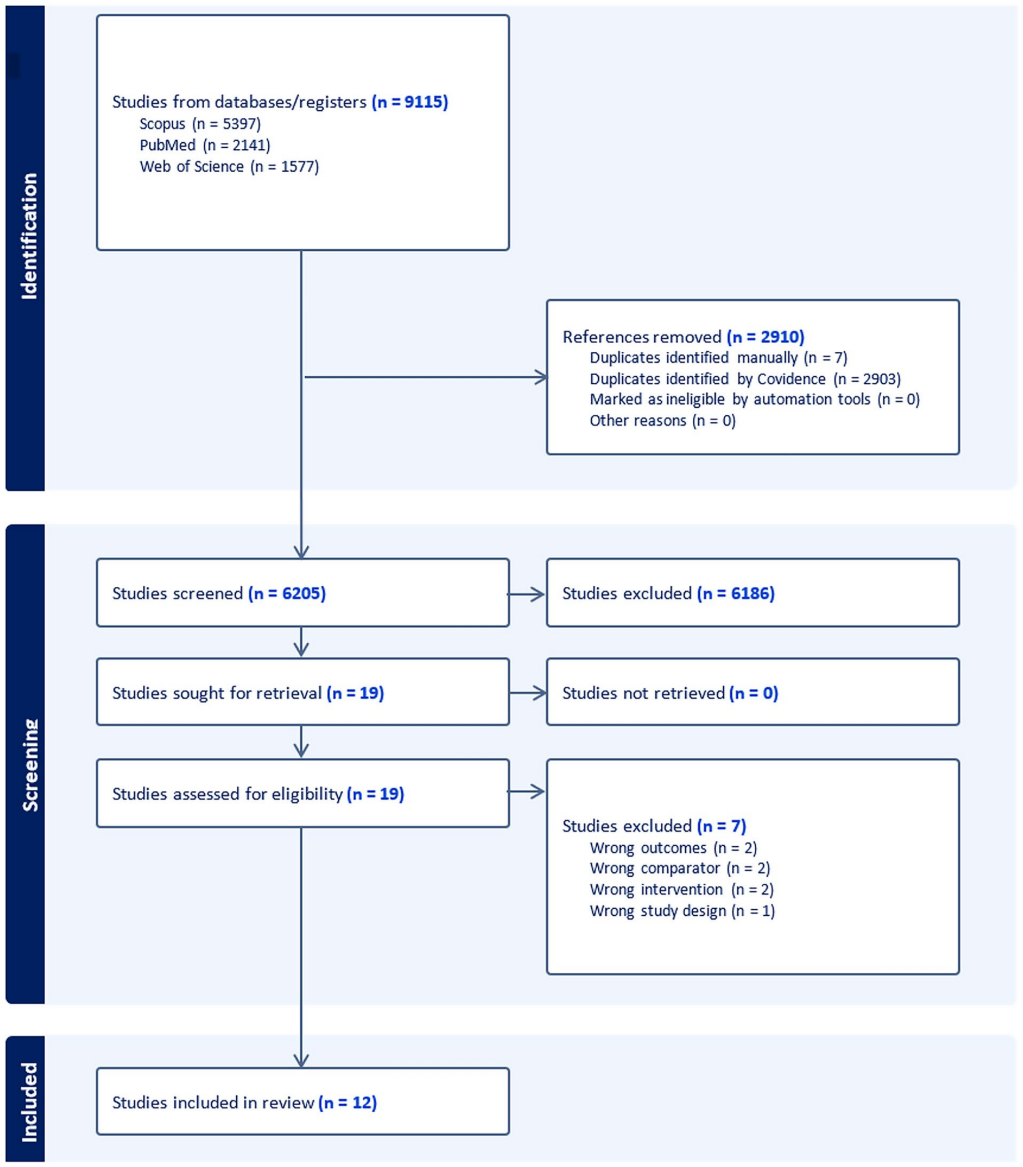
PRISMA flowchart

Nine thousand one hundred and fifteen studies were identified from the literature. Twelve studies [18–29] were found to be eligible following screening (Fig. 1).

### Baseline characteristics

All included studies were written in the English language. The included studies encompassed diverse populations from the United States of America, the United Kingdom, Finland, Korea, and Denmark, with sample sizes ranging from 119 to over 37 million pregnant women. The total sample size was 94,195,776. Most studies focused on migraine during pregnancy, with one investigating all pregnancy-related headaches [21]. The proportion of individuals with migraine or pregnancy-related headaches varied, from 0.18% to 6.3% of the studied pregnant populations. The average maternal age was typically between 28 and 34 years. Parity data, when available, showed a mix of nulliparous and multiparous women, with no consistent pattern across migraine status. Racial and ethnic distributions varied by region but showed a notable predominance of White women in the US and European cohorts, while Asian cohorts were reported in Korean and some US datasets. Follow-up durations ranged from hospitalization-only periods to over a decade in population-based studies. Further information is displayed in Table 1.

**Table 1:** Baseline characteristics

Study ID	Country	Sample Size	Age	Parity	Race/Ethnicity	BMI in kg/m2	Gestational co-morbidities (preeclampsia/eclampsia/GTD/GTH) in %	Previous stroke	Smoking/Alcohol/Drug use	Follow-up duration
Reddy2024	USA	Overall: 19,825,525 Migraineurs: 219,175 (1.1%) Nomigraineurs: 19,506,350 (98.9%)	Age < 18: 259,980 (1.3%) Age 18–34: 15,643,896 (79.3%) Age ≥ 35: 3,582,598 (18.2%)	Multiparous women: 9100	White: 49.7% Black: 15.3% Asian/Pacific Islander: 5.8% Hispanic: 20.1% Other race: 4.4%		Preeclampsia: 11.9% Eclampsia: 0.1% GTH: 5.9%	N/A	Smoking: 961,994(4.9%)	Retrospective National analysis (from hospital records covering 2016 to 2019
Scott2012	UK	Antenatal Stroke cases: 30 Non-hemorrhagic: 18(60%) Hemorrhagic: 12(40%) Control patients: 89	Age younger than 35: 96/119 (80.7%) Age 35 or older: 23/119 (19.3%)	Nulliparous: 51/119 (47.9%) Multiparous: 61/119 (51.3%) Unknown: 1/119 (0.84%)	White: 97/119 (81.5%) Non-white: 19/119 (16.0%) Not known: 3/119 (2.52%)	BMI lower than 30: 97/119 (81.5) BMI 30 or higher: 14/119 (11.8%) Not known: 8/119 (6.72%)	Preeclampsia 8/119 (6.72%) Eclampsia: 2/119 (1.68%) Preeclampsia or eclampsia: 9/119 (7.56%) GTN Diabetes: 5/119 (4.2%)	All cases except one was the first-ever stroke.	Smoker: 24/119 (20.2%)	October 2007- March 2010
James2005	USA	Total Pregnant Patients: 9,135,755 Strokes: 2,850	Of those with stroke: < 20: 290 20–24: 535 25–29: 575 30–34: 697 35–39: 564 >40: 190		Of those with stroke : White; 1078 (37.8%) African American: 435 (15.3%) Hispanic: 356 (12.5%)					Retrospective analysis of national records during the period from 2000 to 2001
Richardt2023	Finland	95 cases and 280 controls	Median (IQR): 30 (9.0)	Median (IQR): 1 (2)		Obesity- BMI greater than or equal to 30 kg/m2: 26/375 (6.9%)	Diabetes during pregnancy: 41/375 (10.9%) GTH: :24/375 (6.4%) Pre-eclampsia 23/375 (6.1%) Postpartum hemorrhage: 7/375 (3.3%)	5/97: 5.15%	Smoking: 55/375 (14.7%)	1987 to 2016
Douglass2021	USA	48 cases of PPCM (43.8% are migraineurs) 96 controls (15.6% are migraineurs)	Mean (SD): 28.0 (7.0) for both cases and controls.	Cases: Nullipara 58.3% Primipara/ Multipara 41.7% Controls Nullipara 44.8% Primipara/ Multipara 55.2%	Cases: White 79.2% American Indian 2.1% Black 18.7% Hispanic 2.1% Controls: White 79.2% American Indian 2.1% Black 18.7% Hispanic 2%	Median (IQR): PPCM 25.2 (20.5–36.6) Controls 23.6 (21.6–28.0)	Cases: GTH: (40%) GTD (4.8%) Controls: GTH: (4.2%) GTD (3.1%)		Cases: Smoking (47.9%) Alcohol (58.7%) Controls: Smoking (43.8%) Alcohol (59.4%)	Cases: 7.2 years (4.1–12.6) Controls: 12.8 years (8.2–18.8)
Bushnell2012	USA	Total: 18 345 538 Migraneurs 33 956 (0.185%) Non migraineurs 18 311 582 (99.815%)	< 20: 0.096% 20–24:0.168% 25–29: 0.231% 30–34: 0.205% 35–39: 0.218% > 40: 0.228% Percentages are derived from rates per 100,000 pregnancy discharges.	N/A	White 0.245% African-American 0.175% Hispanic 0.085% Other 0.086% Missing 0.191% Percentages are derived from rates per 100,000 pregnancy discharges.	N/A	Preeclampsia/ Eclampsia 0.390% GDM: 0.199% GTH: 0.368% Percentages are derived from rates per 100,000 pregnancy discharges.	N/A	489 per 100,000 discharges with migraine were smokers	Analyzed hospital discharges from 2000–2003
Nam2023	Korea	906,187 pregnant women Gestational Headaches: 56,813 No Gestational Headaches: 849,374	Mean (SD) Gestational Headaches: 31.25 (4.27) No Gestational Headaches: 31.73 (4.09)	N/A	N/A	N/A	GTH: 53% GTD: 5.85%	N/A	N/A	Median follow-up period: 8.03 years.
Faden 2016	Canada	4,363,343 pregnancy-related discharges 79 cases of SCAD	SCAD n (%) *n* = 79 (%) < 20: 0 20–24: 5 (6.3) 25–29: 14 (17.7) 30–34: 29 (36.7) 35–39: 18 (22.8) 40–44: 13 (16.5) 45 and more: 0 No SCAD n (%) n= 4 363 264 (%) <;20: 409 541 (9.4) 20–24: 1 044 363 (23.9) 25–29: 1 234 158 (28.3) 30–34: 1 042 051 (23.9) 35–39: 508 562 (11.7) 40–44: 116 014 (11.7) 45 and more: 8575 (0.2)	N/A	SCAD White: 44 (55.7) Black: 18 (22.8) Hispanic: 9 (11.4) Other: 8 (10.1) No SCAD White: 2 535 504 (58.1) Black: 565 374 (13.0) Hispanic: 850 269 (19.5) Other: 412 117 (9.4)	N/A	N/A	N/A	N/A	2008 to 2012
Bandoli2020	USA	Total: 2,892,756 Non-migraineurs 2,866,316 (99.08%) Migraineurs: 26,440 (0.92%)	Migraneurs < 18: 1.2% 18–34: 80.8% > 34: 18% Non- migraineurs < 18: 2.9% 18–34: 79.4% > 34: 17.7%	N/A	Migraineurs: Non-Hispanic white 41.0% Hispanic 34.7% Black 8.8% Asian 5.4% Other 10.1% Non-migraineurs: Non-Hispanic white 25.9% Hispanic 48.9% lack 5.3% Asian 12.55% Other 7.4%	BMI Migraineurs < 25: 48.6% 25-29.9: 29.0% ≥ 30.0: 30.5% Unknown: 6.6% Non- migraineurs < 25: 50.6% 25-29.9: 23.7% ≥ 30.0: 18.9% Unknown: 6.7%	Migraineurs: Hypertension without preeclampsia: 8.0% Preeclampsia: 7.1% Non- migraineurs: Hypertension without preeclampsia: 3.6% Preeclampsia: 3.4%	N/A	Migraineurs Smoking: 8.9% Alcohol: 1.5% Non- migraineurs Smoking: 4.5% Alcohol: 0.4%	The data collection period was five years, from January 2007 to December 2012
Karjalainen2021	Finland	Total: 995 Stroke Cases: 252 Controls without Strokes: 743	Mean (SD): 34.6 (6.15)	Nulliparous: 38.4% primiparous:33.4% Multiparous: 28.3%		Mean (SD): 24.7 (5.4)	GTH: 8% GTD: 11.8% Preeclampsia/eclampsia:5%		12.4%	30 years from 1987 to 2016
Fuglsang2024	Denmark	Total: 1,364,267 Non Migraineurs: 1,307,456 Migraneurs: 56,811	Median (IQR) Median age ranged from 30 to 33 years Non Migraineurs: 30 (27.0–33.6) Migraineurs:31 (28.2–34.7)	Non Migraineurs: 1: 576,320 (44.1%) 2: 467,030 (35.7%) 3–4: 170,407 (13.0%) > 5: 59,448 (4.5%) Missing: 34,251 (2.6%) Migraineurs: 1: 24,072 (42.4%) 2: 20,498 (36.1%) 3–4: 8154 (14.4%) > 5: 2933 (5.2%) Missing: 1154 (2.0%)		Non Migraineurs: Underweight: 34,000 (2.6%) Normal: 489,365 (37.4%) Overweight: 158,827 (12.1%) Obese: 88,426 (6.8%) Missing: 45,043 (3.4%) Migraineurs: Underweight: 1865 (3.3%) Normal: 26,334 (46.4%) Overweight: 9461 (16.7%) Obese: 5555 (9.8%) Missing: 2103 (3.7%) *BMI was not recorded before 2004	Pregnancy Induced hypertension Non Migraineurs: 47,863 (3.7) Migraineurs: 3185 (5.6)	Excluded.	Smoking during pregnancy Non Migraineurs Smoker: 188,501 (14.4) Non Smoker: 991,099 (75.8) Missing: 127,856 (9.8) Migraineurs Smoker: 7373 (13.0) Non smoker: 46,374 (81.6) Missing: 3064 (5.4)	Nationwide population-based longitudinal cohort study on women who gave birth in Denmark during 1996–2018.
Elgendy2020	USA	Total Pregnant Population 37,360,772 Stroke/TIA 16,694	Median (IQR) All Hospitalizations 28 (23–32) Stroke/TIA 30 (25–35)		*All Hospitalizations: White:52.4% Black: 15% Hispanic: 21.7% Asian or Pacific Islander: 5.3% Native American: 0.8% Other: 4.8% *Stroke/TIA White: 45.1% Black: 26.4% Hispanic: 18.3% Asian or Pacific Islander: 4.1% Native American: 0.7% Other: 5.2%	All Hospitalizations: Obesity 4.8% Stroke/TIA: Obesity 7.7%	All Hospitalizations: Gestational hypertension 3.4% Pre-eclampsia/eclampsia 4.4% Gestational diabetes 7.4% Stroke/TIA: Gestational hypertension 2.8% Pre-eclampsia/eclampsia 19.3% Gestational diabetes 6.8%	All Hospitalizations: 0.1% Stroke/TIA: 4%	*All Hospitalizations: Smoking 2.1% Alcohol abuse 0.1% Illicit drug abuse 0.5% *Stroke/TIA: Smoking 7% Alcohol abuse 0.3% Illicit drug abuse 1%	Nationwide retrospective analysis between January 2007 and September 2015.

Abbreviations: GTD, Gestational Diabetes; GTH, Gestational Hypertension; USA, United States of America; UK, United Kingdom; BMI, Body Mass Index; PPCM, Peripartum Cardiomyopathy; N/A, Not Available; IQR, Inter-quartile Range; SD, Standard Deviation; TIA, Transient Ischemic Attack; SCAD, Spontaneous Coronary Artery Dissectoon

### Meta-analysis

Table 2 provides a summary of all effect measures reported in the included studies for each outcome, alongside its adjustment status.

### All strokes and transient ischemic attacks

**Fig. 2:**
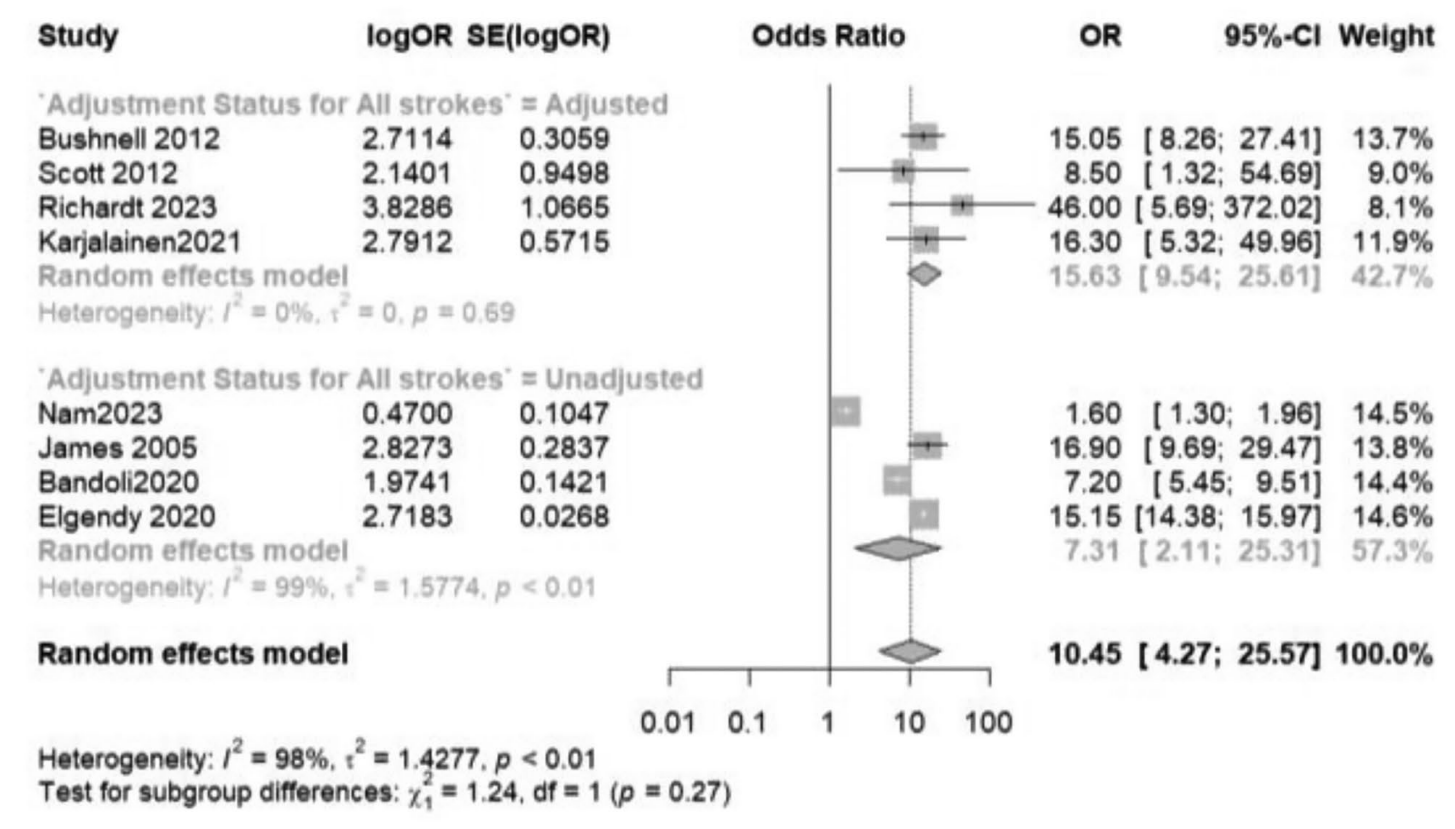
A comparative meta-analysis of the odds of all strokes and transient ischemic attacks

With regards to pregnancy-related headaches, a meta-analysis (Fig. 2) of 8 studies involving 68,642,497 pregnancies revealed an increased odds of 10.45 (95% CI: 4.27 to 25.57) for strokes and TIAs in those with headaches versus those without headaches. These increased odds were observed in both adjusted and unadjusted models. Surprisingly, those studies with adjusted odds ratios resulted in greater odds of strokes and TIAs compared to studies with unadjusted odds ratios (15.63 and 7.31, respectively). A separate analysis of migraine headaches only and their impact on the incidence of strokes and TIAs is shown in the supplementary file.

### Ischemic strokes

**Fig. 3:**
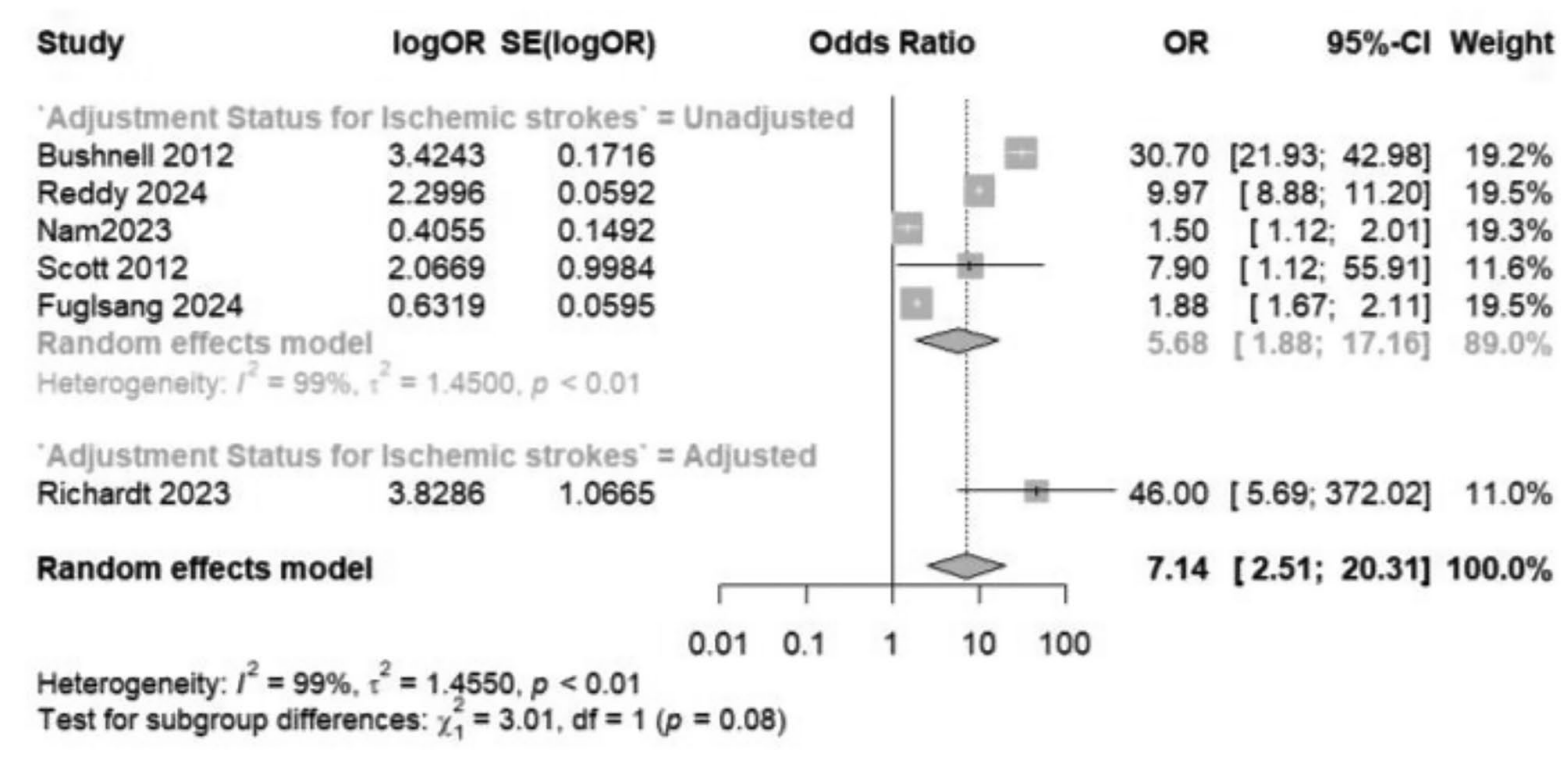
A comparative meta-analysis of the odds of ischemic strokes

Six studies (Fig. 3) reported the odds of ischemic strokes in pregnant women with migraine and pregnancy-related headaches versus those without pregnancy-related headaches; a pooled odds ratio of 7.14 (95% CI: 2.51 to 20.31) was computed for 40,441,999 pregnancies. Evident heterogeneity (I^2^ = 99% and *p* < 0.01) revealed discrepancies in the odds ratios reported in each study, ranging from 46.00 in Richardt et al. [19] to 1.50 in Nam et al. [21]. A separate analysis of migraine headaches only and their impact on the incidence of ischemic strokes is shown in the supplementary file.

### Hemorrhagic strokes

**Fig. 4:**
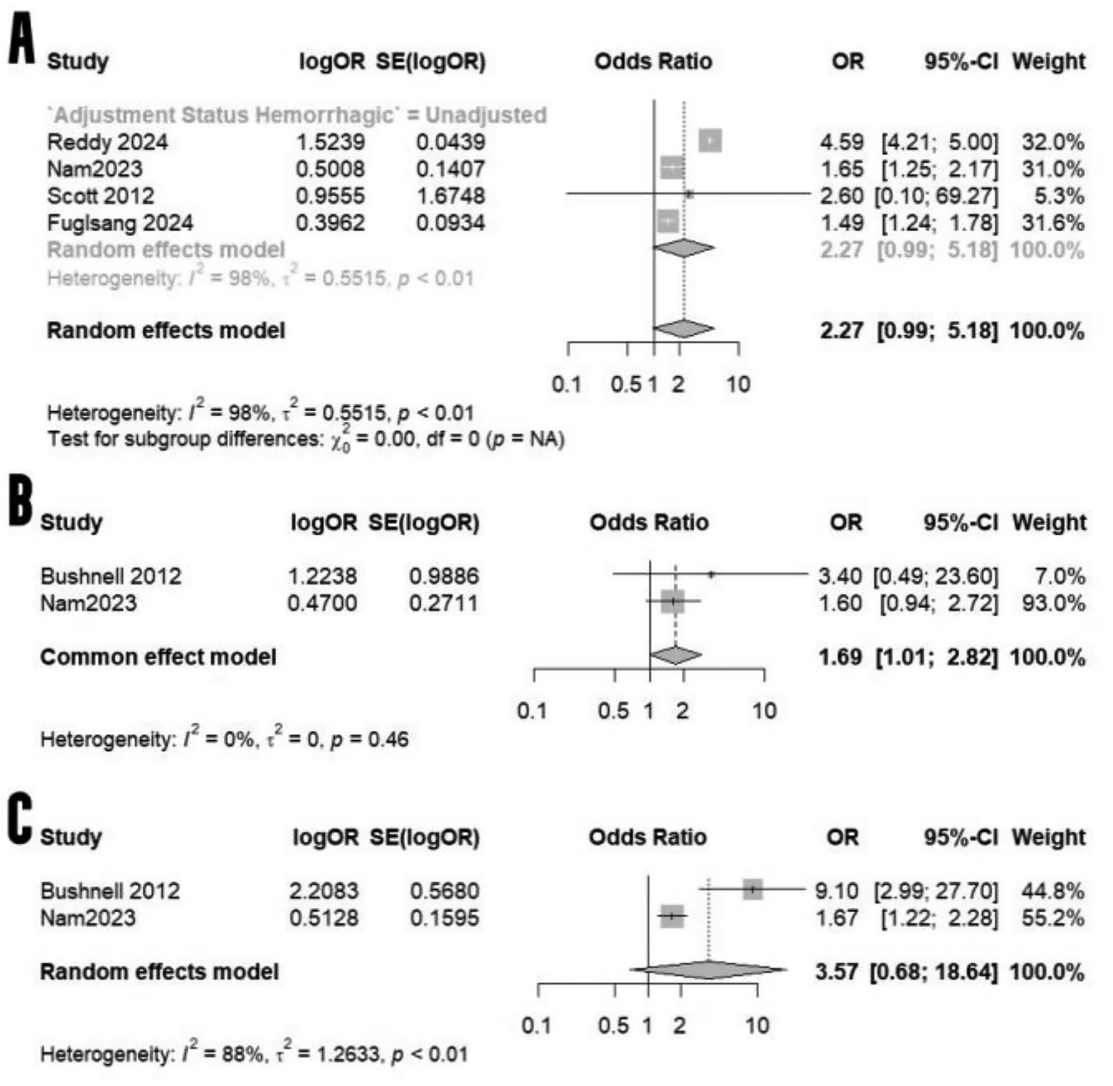
A comparative meta-analysis of the odds of all hemorrhagic strokes **(A)**, subarachnoid hemorrhage **(B)**, and intracranial hemorrhage **(C)**

While the odds of hemorrhagic strokes were comparable among those with pregnancy-related headaches and those without pregnancy-related headaches (95% CI: 0.99 to 5.18, Fig. 4A), a separate analysis of SAH and intracranial hemorrhage ICH in two studies encompassing 19,251,725 pregnancies showed increased odds of SAH in migraineurs and those with pregnancy-related headaches by 69% (Fig. 4B), albeit with an insignificant rise in ICH odds (Fig. 4C). A separate analysis of migraine headaches only and their impact on the incidence of hemorrhagic strokes is shown in the supplementary file.

### Myocardial infarctions, peripartum cardiomyopathy, and coronary dissection

**Fig. 5:**
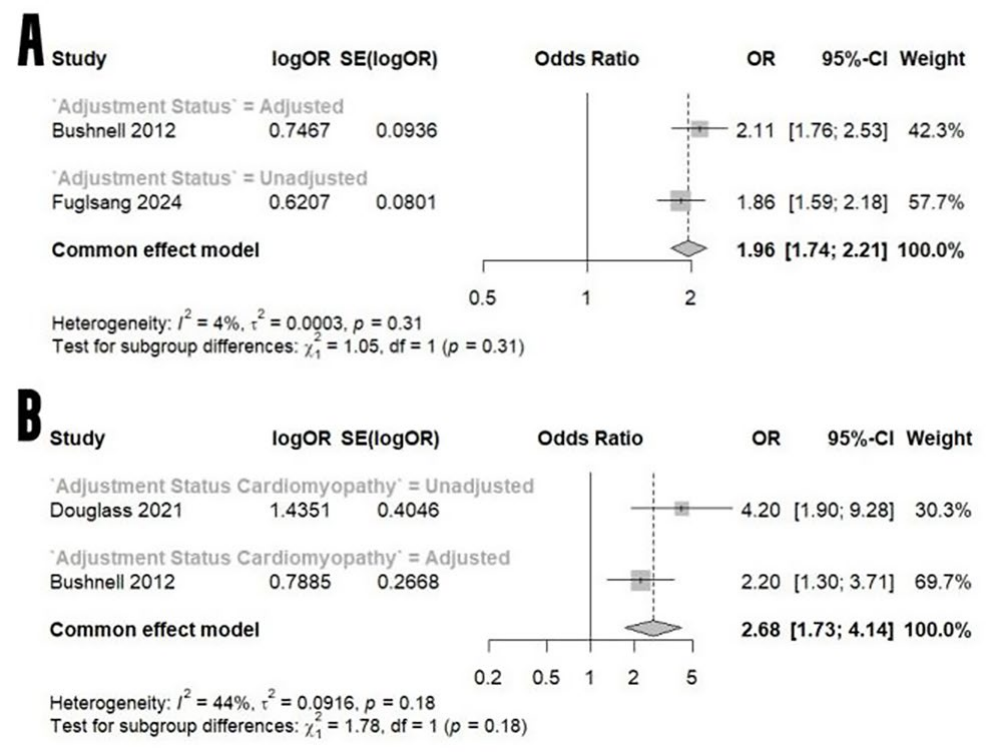
A comparative meta-analysis of the odds of myocardial infarctions **(A)** and cardiomyopathy **(B)**

According to two studies, the odds of myocardial infarction are increased by 96% in pregnant women with pregnancy-related headaches versus those without pregnancy-related headaches (Fig. 5A). With regards to cardiomyopathy, it appears that those with migraine have elevated odds compared to their counterparts without migraine (OR: 2.68, 95% CI: 1.73 to 4.14, Fig. 5B). Faden et al. [29], in their 2016 population-based cohort study of 4,363,343 pregnancies with 79 spontaneous coronary artery dissection (SCAD) cases identified migraine as a significant predictor of SCAD - independent of age, race, or income (adjusted OR: 9.21, 95% CI: 3.72 to 22.82).

### Venous thromboembolic events

A single study by Bushnell et al.^27^ assessed the risk of venous thromboembolism/pulmonary emboli in those with peripartum migraine versus those without. In their multivariate logistic regression model, which adjusted for age and pre-eclampsia diagnoses, migraine were independently associated with increased odds of venous thromboembolism/pulmonary emboli (OR: 3.23, 95% CI: 2.06 to 7.07, p-value: <0.001).

### Quality assessment

All studies were classified as having “good quality” according to the AHRQ standards. Details of each domain in the NOS quality assessment scale are shown in the supplementary file.

**Table 2:** Summary of findings

Study ID	All Strokes		Ischemic Strokes		Hemorrhagic Strokes		Others (myocardial infarctions, peripartum cardiomyopathy, thromboembolic events, all-composite MACCE)	
	Effect estimate, lower CI, Upper CI	If adjusted, then adjusted for what?	Effect estimate, lower CI, Upper CI	If adjusted, then adjusted for what?	Effect estimate, lower CI, Upper CI	If adjusted, then adjusted for what?	Effect estimate, lower CI, Upper CI	If adjusted, then adjusted for what?
Reddy2024			- Unadjusted **OR** (All migraine): **9.97** **(8.88–11.20)** - Migraine with aura: Adjusted **OR 23.26** **(18.46–29.31)** - Migraine without aura: Adjusted **OR 8.15** **(4.79–13.876)**	Adjusted for: Smoking, preeclampsia, eclampsia, thrombophilia, or other disorders requiring ACs/APs, HTN, obesity, COPD, CHF, diabetes mellitus, and OSA.	- Unadjusted **OR** (All migraine): **4.59** **(4.21-5.00)** - Migraine with aura: Adjusted **OR 2.50** **(1.79–3.49)** - Migraine without aura: Adjusted **OR 1.24** **(0.66–2.31)**	Adjusted for: Smoking, preeclampsia, eclampsia, thrombophilia, or other disorders requiring ACs/APs, HTN, obesity, COPD, CHF, diabetes mellitus, and OSA.		
Scott2012	Adjusted**OR: 8.5 (1.5–62.1)**	Maternal age.	**OR: 7.9(1.2–60.1)**	Unadjusted.	**OR: 2.6 (0.05–35.5)**	Unadjusted.		
James2005	**OR 16.9 (9.7–29.5)**	Unadjusted.						
Richardt2023			**OR 46.00(5.69-372.18)**	Age, parity, obesity, smoking, chronic HTN, hypercholesterolemia, diabetes mellitus, gestational hypertension, pre-eclampsia, cesarian section, postpartum hemorrhage				
Douglass 2021							PPCM: **OR 4.2 (1.9–9.28)**	Unadjusted.
Bushnell2012	**OR 15.05 (8.26 to 27.4)**	Adjusted for: Age, pre-eclampsia, venous thromboembolism/pulmonary embolus, acute myocardial infarction/heart disease, hypertension, smoking, diabetes	**OR 30.7 (17.4–34.1)**	Unadjusted.			Myocardial infarction/ heart disease **OR 2.11 (1.76 to 2.54)** Pregnancy-related cerebrovascular event **OR 14.1 (8.7 to** **23.1)** Cardiomyopathy **OR** **2.2 (1.3 to 3.7)**	Myocardial infarction/ heart disease is adjusted for: Age, pre-eclampsia, venous thromboembolism/pulmonary embolism, heart disease, hypertension, smoking, diabetes
Nam2023	**HR: 1.59(1.30–1.95)**	Adjusted for: Age, hypertension, diabetes, functional disability, history of headache, gestational HTN, and gestational diabetes.	**HR: 1.50 (1.12–2.01)**	Adjusted for: Age, hypertension, diabetes, functional disability, history of headache, gestational HTN, and gestational diabetes.	**HR: 1.63 (1.23–2.15)** ICH **HR: 1.63(1.19–2.23)** SAH **HR:1.65 (0.97–2.82)**	Adjusted for: Age, hypertension, diabetes, functional disability, history of headache, gestational hypertension, and gestational diabetes.		
Faden2016							SCAD: Adjusted OR: 9.21, 95% CI: 3.72 to 22.82	Age, race, income
Karjalainen2021	**OR:16.3(5.3–49.8)**	Adjusted for: Maternal age, delivery year, parity, and smoking after 12 weeks.						
Bandoli 2020	**OR** **7.2 (5.5–9.6)**	Unadjusted.						
Fuglsang 2024			0–20 Year Adjusted **HR 1.83 (1.59; 2.10)** 0–10 Year Adjusted **HR 1.75 (1.41; 2.17)**	Adjusted for age, year at delivery, parity, smoking status, and the following comorbidities: HTN, hyperlipidemia, venous thromboembolism, obesity, alcohol-related disease, and kidney disease *0–10 years is restricted to 2004–2018 and includes adjustments for BMI. .	0–20 Year Adjusted **HR** **1.32 (1.06; 1.64)**	Adjusted for age, year at delivery, parity, smoking status, and the following comorbidities: HTN, hyperlipidemia, venous thromboembolism, obesity, alcohol-related disease, and kidney disease.	0–20 Year Myocardial Infarction Adjusted **HR1.60 (1.32; 1.93)** 0–10 Year Myocardial Infarction Adjusted **HR: 1.19 (0.84; 1.68)** 0–20 Year All Premature MACCE Adjusted **HR: 1.65 (1.49; 1.82)** 0–10 Year All Premature MACCE Adjusted **HR: 1.28 (0.92; 1.78)**	Adjusted for age, year at delivery, parity, smoking status, and the following comorbidities: HTN, hyperlipidemia, venous thromboembolism, obesity, alcohol-related disease, and kidney disease. *0–10 years is restricted to 2004–2018 and includes adjustments for BMI.
Elgendy2020	Migraine 0.7% of all hospitalizations Migraine 9.6% of Stroke/TIA (**OR**: **15.2 (14.4–16**)	Unadjusted.						

Abbreviations: CI, Confidence Interval; OR, Odds Ratio; HR, Hazard Ratio; MACCE, Major Adverse Cardiovascular and Cerebrovascular Events; ACs, Anticoagulants; APs, Antiplatelets; HTN, Hypertension; COPD, Chronic Obstructive Pulmonary Disease; CHF, Chronic Heart Failure; OSA, Obstructive Sleep Apnea; PPCM, Peripartum Cardiomyopathy; ICH, Intracerebral Hemorrhage; SAH, Subarachnoid Hemorrhage; BMI, Body Mass Index; TIA, Transient Ischemic Attack

## Discussion

This systematic review and meta-analysis provides robust evidence that migraine is associated with a substantially increased risk of stroke and transient ischemic attack (TIA) during pregnancy, up to 15-fold higher compared with non-migraineurs. Furthermore, migraine appears to be an independent risk factor for cardiovascular events in this population.

Migraine, particularly with aura, is a well-established risk factor for ischemic stroke in young women [5, 6, 11, 30]. Our findings confirm that this elevated risk extends into pregnancy, a period already characterized by a prothrombotic and proinflammatory state [31]. The relevance is heightened by the high prevalence of migraine in women of childbearing age, with up to 40% experiencing a migraine attack by the end of their reproductive years [32, 33]. Physiologic changes during pregnancy, including increased estrogen and progesterone, promote hypercoagulability and venous stasis [34], which, when combined with migraine-related endothelial dysfunction [35], platelet aggregation [36], and vascular dysregulation [37], may synergistically increase vascular risk. Additionally, migraineurs have a higher prevalence of patent foramen ovale (PFO) (an estimated summary OR of 5.13 in a meta-analysis of 1517 patients with PFO) [38], which may serve as a conduit for paradoxical embolism [6], particularly in the setting of deep vein thrombosis - another risk that increases during pregnancy [9]. Thus, the triad of migraine, pregnancy-induced hypercoagulability, and PFO offers a plausible mechanism for ischemic stroke in this population. However, this remains speculative due to the lack of diagnostic detail in most administrative datasets.

Due to the paucity of evidence, we were unable to stratify migraineurs’ risk of cardiovascular and cerebrovascular events according to the aura. Nevertheless, as displayed in Table 2, the retrospective record-based national analysis of pregnant women by Reddy et al.^20^ included 19,825,525 pregnant women - of which 219,175 were with migraine - and concluded that migraine with aura heightened the odds of ischemic strokes by 23 times compared to 8 times in those without aura. The evidence for a greater risk of cerebrovascular events in those with aura is reinforced by a 2022 meta-analysis of 12 studies; it computed a greater hazard ratio of ischemic strokes in those with aura (1.4) compared to migraineurs without aura (1.1)^11^. However, their analysis did not include pregnant women; therefore, such an association of migraine with auras with a greater risk of ischemic strokes cannot be extrapolated to the pregnant women population as of the moment without further prospective studies - apart from Reddy et al.^20^ Several factors can explain this discrepancy in the incidence of ischemic strokes: Studies suggest a greater incidence of PFO in migraine with aura, estimated to be around 46.3–88% in migraine patients with aura compared with 16.2–34.9% in migraine patients without aura [39]. Furthermore, cortical spreading depression is a hallmark of migraine with aura, but is a contested concept in migraine without aura [40]. It leads to transient oligemia followed by reactive hyperemia, which can result in localized hypoxia, disruption of the blood-brain barrier, and activation of inflammatory pathways [41]. These pathophysiological changes increase the vulnerability of cerebral tissue to ischemic events [41]. In contrast, evidence of this spreading depression is inconclusive in migraine without auras, thereby reducing the burden of such vascular stressors.

The association between migraine and hemorrhagic stroke also warrants attention. Migraine - through mechanisms such as cortical spreading depression [40] and reversible cerebral vasoconstriction syndrome (RCVS) [42] - can compromise cerebral autoregulation and predispose to vessel wall damage. These pathophysiological mechanisms, compounded by the hemodynamic changes of pregnancy, may contribute to the heightened risk of SAH shown in our analysis. While overall hemorrhagic stroke risk was not significantly elevated, SAH odds were elevated by 69% among migraineurs, whereas ICH odds were not in our analysis. This disparity may be explained by pathophysiological differences: SAH typically results from rupture of superficial arterial aneurysms [43], potentially exacerbated by migraine-associated vascular instability and pregnancy-related hemodynamic stress - while ICH is usually related to small-vessel hypertensive disease [44], which migraine may not directly influence. RCVS is more commonly associated with SAH in the literature [45], as illustrated in Ducros et al.‘s [46] analysis of prospective data for 89 consecutive patients with RCVS, 30 of which developed at least one type of ICH. These included 27 cases of cortical SAH, compared to 11 cases of intracerebral hemorrhage. Of interest, both female gender and history of migraine were associated with significantly greater risk of ICH (OR: 4.05 and 2.34, respectively).

Another consideration is the overlap between migraine and hypertensive disorders of pregnancy, especially preeclampsia. Both conditions share features such as headache, endothelial dysfunction, and elevated blood pressure [47]. Administrative data may not reliably distinguish between migraine and severe preeclampsia, leading to potential misclassification bias. However, this overlap could also indicate a shared pathophysiological pathway, particularly as both conditions have been linked to increased blood pressure during pregnancy and to other vascular complications such as posterior reversible encephalopathy syndrome [48] and RCVS [49]. Bushnell et al.^27^ argued against the notion of a misrepresentation of the impact migraine play in triggering stroke events in pregnancy. Following the exclusion of any overlapping pre-eclampsia codes, a 15-fold increased risk of strokes was revealed in pregnant women with migraine. Realistically, it could be that multiple pathophysiological links interplay to produce the demonstrated risk of vascular events in pregnant women with migraine. Bandoli et al.^28^ estimated that approximately 25% of stroke events in their cohort of 26,440 pregnant women with migraine could be attributed to hypertensive disorders, leaving the remaining 75% of events open to pathophysiologic discussions.

It was interesting to note how migraine emerged as a significant risk factor for cardiovascular events - such as myocardial infarctions and peripartum cardiomyopathies - in this meta-analysis. Migraine has been previously proposed as a “systemic vasculopathy" [50] that may impact non-cerebral blood vessels. This is supported by evidence of peripheral arterial and endothelial dysfunction in migraineurs [51]. A 2013 systematic review of eight studies reporting on arterial function revealed that indices such as pulse wave velocity and augmentation index are elevated in migraineurs, indicating increased peripheral arterial stiffness and disrupted compliance mechanisms [51]. This concept of arterial dysfunction in migraine is further supported by the evidence of increased odds of arterial dissection in those with a history of migraine. The multicenter Italian Project on Stroke in Young Adults identified significantly greater odds of migraine history in those with cervical arterial dissection. Of the 334 patients with dissection, 103 had a positive history of migraine, and of the 2151 ischemic stroke patients without dissection, 525 had a history of migraine attacks (103 [30.8%] vs. 525 [24.4%], *P* = 0.01) [52].

Interestingly, Nam et al. [21] found that pregnancy-related headaches without prior headache history were not associated with increased vascular risk, whereas women with both a headache history and new-onset pregnancy headaches had a five-fold increased risk of ischemic stroke postpartum. This suggests that chronic headache disorders persisting into pregnancy may confer greater vascular vulnerability and higher risk of complications than de novo pregnancy headaches. Another point worth mentioning is how our analysis did not correlate the underlying headache characteristics (such as intensity, frequency, and duration) with the subsequent risk of MACCE. It has been predicted by Ashrafinia et al. [53] in a 400-pregnant-women cohort that the headache intensity before pregnancy can significantly impact headaches during pregnancy. Therefore, to better inform clinicians on the stratified risk of MACCE, such granular headache characteristics need to be available both pre- and intrapartum, effectively helping in the creation of tailored approaches for each patient based on their expected risk of developing MACCE.

Emerging evidence suggests that misconceptions about migraine and its treatment significantly influence reproductive decisions. Recent Japanese surveys [54, 55] showed that up to 5.4% of women avoided pregnancy because of headaches, particularly migraine, due to concerns about disability, medication safety, and potential harm to the child. Women in the “avoid pregnancy” groups not only experienced a higher headache burden but also demonstrated lower awareness of headache-related facts, highlighting the impact of limited education and persistent stigma. From this standpoint, we stress the need for tailored counseling for young women in their reproductive period to dispel misconceptions, emphasize safe treatment options, and raise awareness of vascular risks during pregnancy. Similarly, clinician education is essential to promote early recognition and evidence-based management of cardiovascular complications in this population. By addressing these educational gaps, healthcare systems will not only improve reproductive autonomy and reduce unnecessary pregnancy avoidance, but they will also mitigate adverse maternal cardiovascular outcomes.

From a clinical perspective, our findings underscore the need for increased scrutiny and targeted counseling for pregnant women with a history of migraine, especially those with aura. On ground measures may include primary care physicians monitoring for hypertensive disorders, familiarizing patients with warning signs of stroke, and considering the early referral to maternal-fetal medicine or neurologists whenever appropriate. While the absolute risk of stroke remains low, we believe that the potential consequences for the mother justify a proactive approach.

### Strengths and limitations

The strengths of this meta-analysis include its large, aggregated sample size, focus on pregnant women (a vulnerable population for major cerebrovascular and thromboembolic events), its use of adjusted effect measures (whenever possible), and the inclusion of multiple vascular outcomes.

Despite the consistency of our findings with the existing literature, several limitations should be acknowledged. Most included studies relied on a retrospective review of administrative or self-reported data, which may introduce coding inaccuracies and underreporting, particularly for less severe or undiagnosed migraine. The possibility of miscoding severe preeclampsia as migraine in non-validated nationwide inpatient studies remains a concern, potentially overestimating the odds of ischemic strokes in migraineurs. Additionally, migraine subtype (aura vs. non-aura), frequency of attacks, temporality (whether migraine was newly diagnosed during pregnancy, or the woman was already diagnosed with migraine before pregnancy), and treatment regimens were not consistently available. Whether or not those receiving medications for migraine prophylaxis and the impact of different treatment options for migraine may positively impact the odds of cerebrovascular/cardiovascular events in pregnancy remains unknown, too. Heterogeneity in the included studies may also stem from variations in population demographics, diagnostic methods, and outcome ascertainment. Although we used adjusted effect estimates where possible, residual confounding cannot be excluded, particularly with respect to cardiovascular comorbidities, medication use (e.g., triptans or ergotamines), and socioeconomic factors.

## Conclusions

In conclusion, this meta-analysis reinforces the notion that pregnancy-related headaches, including migraine, are independent risk factors for ischemic stroke and SAH during pregnancy - given the elevated odds in adjusted models. In addition, an analysis of myocardial infarction and cardiomyopathy – including adjusted and unadjusted models – showcased the elevated odds in pregnant women with headaches. Venous thromboembolic events and SCAD events significantly rise in migraineurs, as demonstrated by two separate studies. It has revealed the strength and significance of the association between migraine and cerebrovascular/cardiovascular events in an already particularly vulnerable population. Future prospective studies with granular clinical detail and more comprehensive phenotyping - including migraine subtype, attack frequency, temporality, and vascular biomarkers - are essential to clarify causal mechanisms and identify women at the highest risk. Such efforts could pave the way for individualized risk stratification and preventative strategies in this vulnerable population. Moreover, mechanistic research investigating hormonal, vascular, and genetic contributors may ultimately enable individualized risk stratification and targeted preventive strategies in this vulnerable population.

## Supplementary Information

Below is the link to the electronic supplementary material.

Supplementary Material 1

## Data Availability

The dataset(s) supporting the conclusions of this article is(are) included within the article (and its additional file(s)).

## Abbreviations

PRISMA: Preferred Reporting Items for Systematic Reviews and Meta-analyses
OR: Odds Ratio
HR: Hazard Ratio
MACCE: Major Adverse Cardiovascular and Cerebrovascular Events
ASCVD: Atherosclerotic Cardiovascular Disease
TIA: Transient Ischemic Attack
CI: Confidence Interval
ICH: Intracerebral Hemorrhage
SAH: Subarachnoid Hemorrhage
NOS: Newcastle-Ottawa Scale
QA: Quality Assessment
BMI: Body Mass Index
USA: United States of America
UK: United Kingdom
SCAD: Spontaneous Coronary Artery Dissection
PFO: Patent Foramen Ovale
