# Supplementary material for: Migraine and pregnancy-related headaches as a risk factor for cardiovascular and cerebrovascular events in pregnancy: a systematic review and meta-analysis of over 94 million pregnancies
Source: J Headache Pain. 2025 Nov 13;26(1):259. doi: 10.1186/s10194-025-02190-1 (PMC12616990; doi:10.1186/s10194-025-02190-1)
Supplement: Supplementary file 1 — Supplementary Material 1 [file 10194_2025_2190_MOESM1_ESM.docx]

**Search Strategy:**

(Migrain* OR headache* OR "Disorder, Migraine" OR "Disorders, Migraine" OR "Migraine Disorder" OR "Headache, Migraine" OR "Headaches, Migraine" OR "Migraine Headaches" OR "Migraine" OR "Migraines" OR "Migraine Headache" OR "Acute Confusional Migraine" OR "Acute Confusional Migraines" OR "Migraine, Acute Confusional" OR "Migraines, Acute Confusional" OR "Status Migrainosus" OR "Abdominal Migraine" OR "Abdominal Migraines" OR "Migraine, Abdominal" OR "Migraines, Abdominal" OR "Cervical Migraine Syndrome" OR "Cervical Migraine Syndromes" OR "Migraine Syndrome, Cervical" OR "Migraine Syndromes, Cervical" OR "Hemicrania Migraine" OR "Hemicrania Migraines" OR "Migraine, Hemicrania" OR "Migraines, Hemicrania" OR "Migraine Variant" OR "Migraine Variants" OR "Variant, Migraine" OR "Variants, Migraine" OR "Sick Headache" OR "Headache, Sick" OR "Headaches, Sick" OR "Sick Headaches" OR "Headaches" OR "Cephalalgia" OR "Cephalalgias" OR "Cephalgia" OR "Cephalgias" OR "Cephalodynia" OR "Cephalodynias" OR "Cranial Pain" OR "Cranial Pains" OR "Pain, Cranial" OR "Pains, Cranial" OR "Head Pain" OR "Head Pains" OR "Pain, Head" OR "Pains, Head" OR "Bilateral Headache" OR "Bilateral Headaches" OR "Headache, Bilateral" OR "Headaches, Bilateral" OR "Generalized Headache" OR "Generalized Headaches" OR "Headache, Generalized" OR "Headaches, Generalized" OR "Ocular Headache" OR "Headache, Ocular" OR "Headaches, Ocular" OR "Ocular Headaches" OR "Orthostatic Headache" OR "Headache, Orthostatic" OR "Headaches, Orthostatic" OR "Orthostatic Headaches" OR "Periorbital Headache" OR "Headache, Periorbital" OR "Headaches, Periorbital" OR "Periorbital Headaches" OR "Retro-Ocular Headache" OR "Headache, Retro-Ocular" OR "Headaches, Retro-Ocular" OR "Retro Ocular Headache" OR "Retro-Ocular Headaches" OR "Sharp Headache" OR "Headache, Sharp" OR "Headaches, Sharp" OR "Sharp Headaches" OR "Throbbing Headache" OR "Headaches, Throbbing" OR "Headache, Throbbing" OR "Throbbing Headaches" OR "Unilateral Headache" OR "Headaches, Unilateral" OR "Headache, Unilateral" OR "Unilateral Headaches" OR "Hemicrania" OR "Vertex Headache" OR "Headaches, Vertex" OR "Headache, Vertex" OR "Vertex Headaches")

**AND**

(pregnan* OR Puerperium OR Peripartum OR Prenatal OR Postnatal OR Postpartum OR Intrapartum OR Antepartum OR Partum OR gestatio*)

**AND**

(stroke* OR cardiovascular OR cerebrovascular OR myocardial OR infarction OR “Cerebral Venous Thrombosis” OR “Cerebral Venous Sinus Thrombosis” OR coronary OR “Transient Ischemic Attack” OR “intracerebral hemorrhage” OR “Subarachnoid Hemorrhage” OR "Strokes" OR "Cerebrovascular Accident" OR "Cerebrovascular Accidents" OR "Cerebral Stroke" OR "Cerebral Strokes" OR "Stroke, Cerebral" OR "Strokes, Cerebral" OR "Cerebrovascular Apoplexy" OR "Apoplexy, Cerebrovascular" OR "Vascular Accident, Brain" OR "Brain Vascular Accident" OR "Brain Vascular Accidents" OR "Vascular Accidents, Brain" OR "Cerebrovascular Stroke" OR "Cerebrovascular Strokes" OR "Stroke, Cerebrovascular" OR "Strokes, Cerebrovascular" OR "Apoplexy" OR "CVA (Cerebrovascular Accident)" OR "CVAs (Cerebrovascular Accident)" OR "Stroke, Acute" OR "Acute Stroke" OR "Acute Strokes" OR "Strokes, Acute" OR "Cerebrovascular Accident, Acute" OR "Acute Cerebrovascular Accident" OR "Acute Cerebrovascular Accidents" OR "Cerebrovascular Accidents, Acute" OR "Infarction, Myocardial" OR "Infarctions, Myocardial" OR "Myocardial Infarctions" OR "Heart Attack" OR "Heart Attacks" OR "Myocardial Infarct*" OR "Infarct, Myocardial" OR "Infarcts, Myocardial" OR "Myocardial Infarcts" OR "Cardiovascular Stroke" OR "Cardiovascular Strokes" OR "Stroke, Cardiovascular" OR "Strokes, Cardiovascular" OR "Cardiovascular Disease" OR "Disease, Cardiovascular" OR "Cardiac Events" OR "Cardiac Event" OR "Event, Cardiac" OR "Adverse Cardiac Event" OR "Adverse Cardiac Events" OR "Cardiac Event, Adverse" OR "Cardiac Events, Adverse" OR "Major Adverse Cardiac Events" OR "Intracranial Sinus Thromboses" OR "Intracranial Sinus Thrombosis" OR "Sinus Thromboses, Intracranial" OR "Thromboses, Intracranial Sinus" OR "Thrombosis, Intracranial Sinus" OR "Cranial Sinus Thrombosis" OR "Cranial Sinus Thromboses" OR "Sinus Thromboses, Cranial" OR "Sinus Thrombosis, Cranial" OR "Thromboses, Cranial Sinus" OR "Thrombosis, Cranial Sinus" OR "Sinus Thrombosis" OR "Sinus Thromboses" OR "Thromboses, Sinus" OR "Thrombosis, Sinus" OR "Venous Sinus Thrombosis, Cranial" OR "Intracranial Sinus Thrombophlebitis" OR "Intracranial Sinus Thrombophlebitides" OR "Sinus Thrombophlebitides, Intracranial" OR "Sinus Thrombophlebitis, Intracranial" OR "Thrombophlebitides, Intracranial Sinus" OR "Thrombophlebitis, Intracranial Sinus" OR "Petrous Sinus Thrombosis" OR "Petrous Sinus Thromboses" OR "Sinus Thromboses, Petrous" OR "Sinus Thrombosis, Petrous" OR "Thromboses, Petrous Sinus" OR "Thrombosis, Petrous Sinus" OR "Petrous Sinus Thrombophlebitis" OR "Petrous Sinus Thrombophlebitides" OR "Sinus Thrombophlebitides, Petrous" OR "Sinus Thrombophlebitis, Petrous" OR "Thrombophlebitides, Petrous Sinus" OR "Thrombophlebitis, Petrous Sinus" OR "Hemorrhage, Subarachnoid" OR "Hemorrhages, Subarachnoid" OR "Subarachnoid Hemorrhages" OR "SAH (Subarachnoid Hemorrhage)" OR "SAHs (Subarachnoid Hemorrhage)" OR "Perinatal Subarachnoid Hemorrhage" OR "Hemorrhage, Perinatal Subarachnoid" OR "Hemorrhages, Perinatal Subarachnoid" OR "Perinatal Subarachnoid Hemorrhages" OR "Subarachnoid Hemorrhage, Perinatal" OR "Subarachnoid Hemorrhages, Perinatal" OR "Subarachnoid Hemorrhage, Aneurysmal" OR "Aneurysmal Subarachnoid Hemorrhage" OR "Aneurysmal Subarachnoid Hemorrhages" OR "Hemorrhage, Aneurysmal Subarachnoid" OR "Hemorrhages, Aneurysmal Subarachnoid" OR "Subarachnoid Hemorrhages, Aneurysmal" OR "Subarachnoid Hemorrhage, Spontaneous" OR "Hemorrhage, Spontaneous Subarachnoid" OR "Hemorrhages, Spontaneous Subarachnoid" OR "Spontaneous Subarachnoid Hemorrhage" OR "Spontaneous Subarachnoid Hemorrhages" OR "Subarachnoid Hemorrhages, Spontaneous" OR "Subarachnoid Hemorrhage, Intracranial" OR "Hemorrhage, Intracranial Subarachnoid" OR "Hemorrhages, Intracranial Subarachnoid" OR "Intracranial Subarachnoid Hemorrhage" OR "Intracranial Subarachnoid Hemorrhages" OR "Subarachnoid Hemorrhages, Intracranial" OR "Brain TIA" OR "TIA, Brain" OR "TIA (Transient Ischemic Attack)" OR "TIAs (Transient Ischemic Attack)" OR "Transient Ischemic Attack" OR "Attacks, Transient Ischemic" OR "Attack, Transient Ischemic" OR "Ischemic Attacks, Transient" OR "Transient Ischemic Attacks" OR "Cerebral Ischemia, Transient" OR "Cerebral Ischemias, Transient" OR "Ischemias, Transient Cerebral" OR "Ischemia, Transient Cerebral" OR "Transient Cerebral Ischemia" OR "Transient Cerebral Ischemias" OR "Brain Stem Ischemia, Transient" OR "Transient Ischemic Attack, Brainstem" OR "Transient Ischemic Attack, Brain Stem" OR "Brain Stem Transient Ischemic Attack" OR "Brainstem Ischemia, Transient" OR "Brainstem Ischemias, Transient" OR "Ischemias, Transient Brainstem" OR "Ischemia, Transient Brainstem" OR "Transient Brainstem Ischemia" OR "Brainstem Transient Ischemic Attack" OR "Crescendo Transient Ischemic Attacks" OR "Transient Ischemic Attacks, Crescendo" OR "Carotid Circulation Transient Ischemic Attack" OR "Transient Ischemic Attack, Carotid Circulation" OR "Posterior Circulation Transient Ischemic Attack" OR "Transient Ischemic Attack, Posterior Circulation" OR "Transient Ischemic Attack, Anterior Circulation" OR "Anterior Circulation Transient Ischemic Attack" OR "Transient Ischemic Attack, Vertebrobasilar Circulation" OR "Vertebrobasilar Circulation Transient Ischemic Attack" OR "Anginas, Unstable" OR "Unstable Anginas" OR "Angina at Rest" OR "Angina, Preinfarction" OR "Anginas, Preinfarction" OR "Preinfarction Angina" OR "Preinfarction Anginas" OR "Unstable Angina" OR "Angina Pectoris, Unstable" OR "Angina Pectori, Unstable" OR "Unstable Angina Pectori" OR "Unstable Angina Pectoris" OR "Myocardial Preinfarction Syndrome" OR "Myocardial Preinfarction Syndromes" OR "Preinfarction Syndrome, Myocardial" OR "Preinfarction Syndromes, Myocardial" OR "Syndrome, Myocardial Preinfarction" OR "Syndromes, Myocardial Preinfarction" OR emboli* OR thromboemboli* OR "Phlebothrombosis" OR "Phlebothromboses" OR "Thrombosis, Venous" OR "Thromboses, Venous" OR "Venous Thromboses" OR "Deep Vein Thrombosis" OR "Deep Vein Thromboses" OR "Thromboses, Deep Vein" OR "Vein Thromboses, Deep" OR "Vein Thrombosis, Deep" OR "Deep Venous Thrombosis" OR "Deep Venous Thromboses" OR "Thromboses, Deep Venous" OR "Thrombosis, Deep Venous" OR "Venous Thromboses, Deep" OR "Venous Thrombosis, Deep" OR "Deep-Vein Thrombosis" OR "Deep-Vein Thromboses" OR "Thromboses, Deep-Vein" OR "Thrombosis, Deep-Vein" OR "Deep-Venous Thrombosis" OR "Deep-Venous Thromboses" OR "Thromboses, Deep-Venous" OR "Thrombosis, Deep-Venous" OR thromb* OR dissect*)

For studies reporting on migraine headaches only, the following analyses were conducted:

**All Strokes and TIAs**

The pooled odds of stroke and TIA events in pregnant migraineurs versus pregnant non-migraineurs were elevated (OR: 13.17, 95% CI: 8.90 to 19.51). This significant elevation was observed in both adjusted and non-adjusted models, as shown in **Fig. S1.**

**
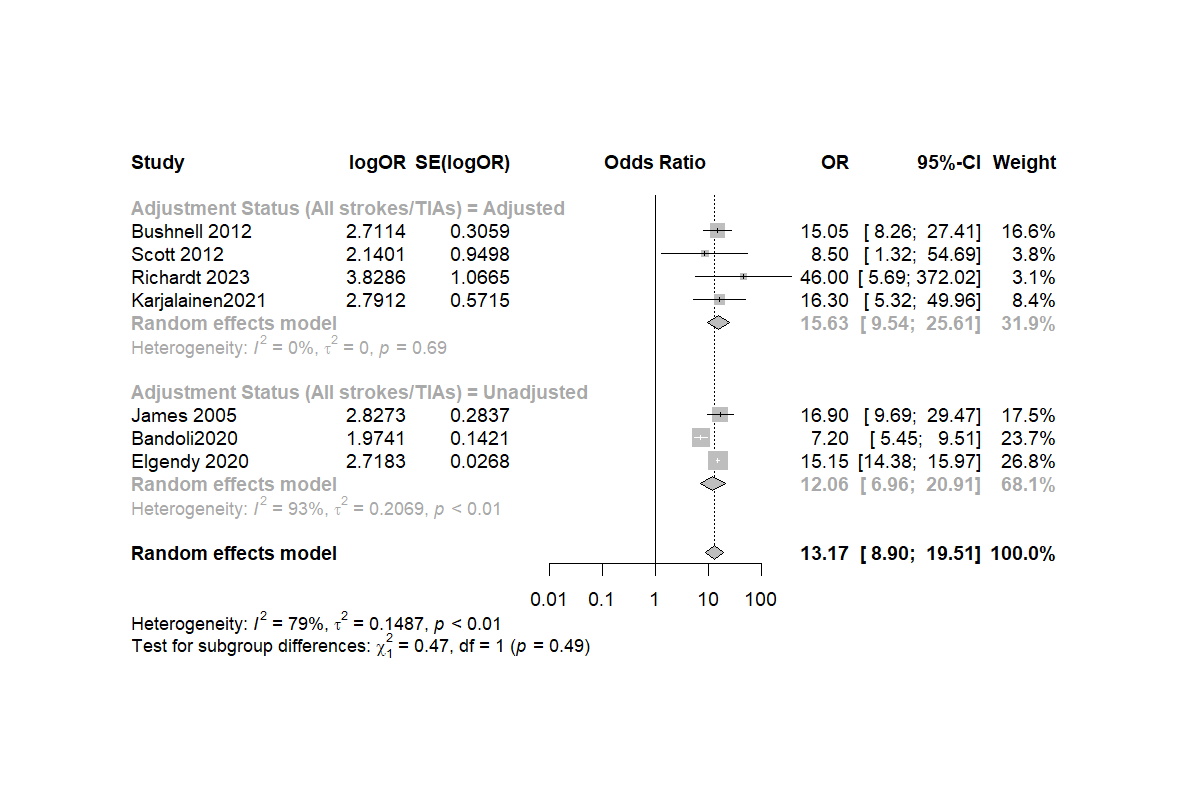
**

**Fig. S1**

**Ischemic Strokes**

The pooled odds of ischemic strokes in pregnant migraineurs versus pregnant non-migraineurs were elevated (OR: 10.41, 95% CI: 3.15 to 34.39). This significant elevation persisted in both adjusted and non-adjusted models, as shown in **Fig. S2.**

**
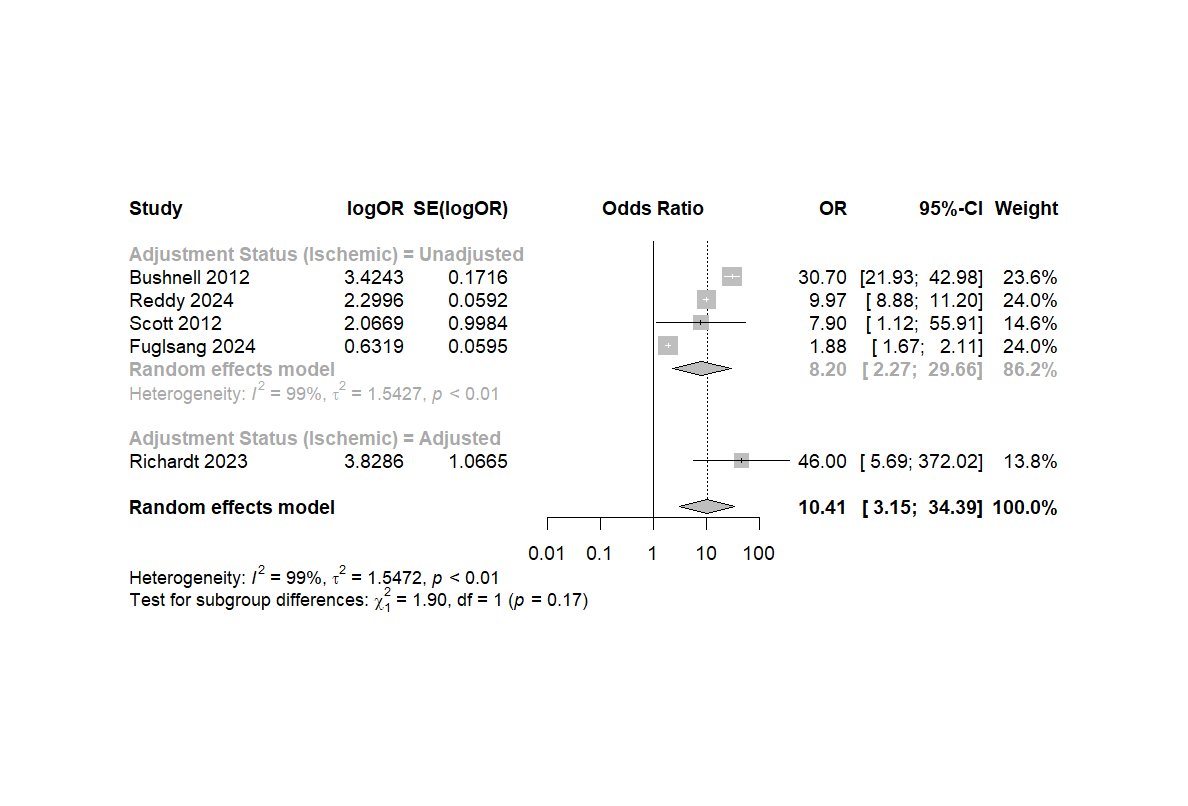
**

**Fig. S2**

**Hemorrhagic Strokes**

Similar to the analysis of all pregnancy-related headaches, no significant association was established between migraine during pregnancy and the incidence of hemorrhagic strokes (OR: 2.62, 95% CI: 0.91 to 7.49).


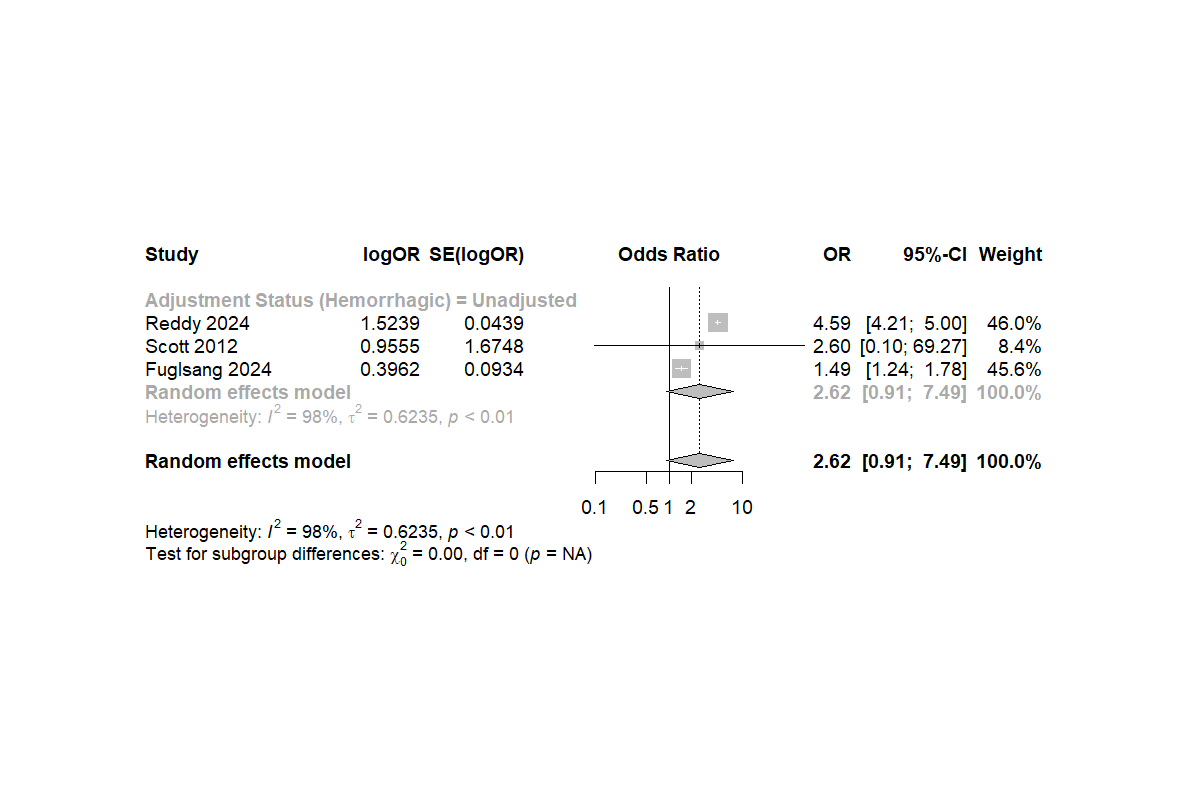


**Fig. S3**

For Cohort Studies

**Table S1:** Quality Assessment

| **Author, Year** | **SELECTION** | | | | **COMPARABILITY** | **OUTCOMES** | | | **Total points/9** | **AHRQ Standardised Rating** |
| --- | --- | --- | --- | --- | --- | --- | --- | --- | --- | --- |
|  | **1) Representativeness of the exposed cohort** | **2) Selection of the non-exposed cohort** | **3) Ascertainment of exposure** | **4) Demonstration that outcome of interest was not present at start of study** | **1) Comparability of cohorts on the basis of the design or analysis** | **1) Assessment of outcome** | **2) Was follow-up long enough for outcomes to occur** | **3) Adequacy of follow up of cohorts** |  |  |
| **Reddy2024** | 1 | 1 | 1 | 0 | 2 | 1 | 1 | 1 | **8** | **Good** |
| **Scott2012** | 1 | 1 | 1 | 1 | 1 | 1 | 0 | 1 | **7** | **Good** |
| **James2005** | 1 | 1 | 1 | 0 | 1 | 1 | 0 | 1 | **6** | **Good** |
| **Richardt2023** | 1 | 1 | 0 | 1 | 2 | 1 | 1 | 1 | **8** | **Good** |
| **Nam2023** | 1 | 1 | 1 | 1 | 2 | 1 | 1 | 1 | **9** | **Good** |
| **Karjalainen2021** | 1 | 1 | 1 | 1 | 2 | 1 | 1 | 1 | **9** | **Good** |
| **Bandoli2020** | 1 | 1 | 1 | 0 | 2 | 1 | 1 | 0 | **7** | **Good** |
| **Fuglsang2024** | 1 | 1 | 1 | 1 | 2 | 1 | 1 | 0 | **8** | **Good** |
| **Elgendy2020** | 1 | 1 | 1 | 0 | 1 | 1 | 1 | 1 | **7** | **Good** |
| **Faden2016** | 1 | 1 | 1 | 1 | 2 | 1 |  | 1 |  |  |

For Case-Control Studies

**Table S2:** NOS Quality Assessment

| **Author, Year** | **SELECTION** | | | | **COMPARABILITY** | **EXPOSURE** | | | **Total points/9** | **AHRQ Standardised Rating** |
| --- | --- | --- | --- | --- | --- | --- | --- | --- | --- | --- |
|  | **1) Is the case definition adequate?** | **2) Representativeness of the cases** | **3) Selection of Controls** | **4) Definition of Controls** | **1) Comparability of cohorts on the basis of the design or analysis** | **1) Ascertainment of Exposure** | **1) Same method of ascertainment for cases and controls** | **2) Non-**  **Response rate** |  |  |
| **Douglass2021** | 1 | 1 | 1 | 1 | 2 | 1 | 1 | 1 | **9** | **Good** |
| **Bushnell 2012** | 1 | 1 | 1 | 1 | 2 | 0 | 1 | 1 | **8** | **Good** |
